# Sulfate sensitivity of early life stages of freshwater mussels *Unio crassus* and *Margaritifera margaritifera*

**DOI:** 10.1007/s10646-024-02794-4

**Published:** 2024-08-08

**Authors:** Xiaoxuan Hu, Mikko Mäkinen, Jouni Taskinen, Juha Karjalainen

**Affiliations:** University of Jyväskylä, Department of Biological and Environmental Science, Jyväskylä, Finland

**Keywords:** Effective concentration, Bivalve, Glochidia, Osmotic stress, Salinity, Toxicity test

## Abstract

Sulfate is increasingly found in elevated concentrations in freshwater ecosystems due to anthropogenic activities. Chronic exposure to sulfate has been reported to cause sublethal effects on freshwater invertebrates. Previous sulfate toxicity tests have mostly been conducted in hard or moderately hard waters, and research on species inhabiting soft water is needed, given that freshwater organisms face heightened sensitivity to toxicants in water of lower hardness. In the present study, we examined sulfate sensitivity of two endangered freshwater mussel species, *Unio crassus*, and *Margaritifera margaritifera*. Glochidia and juveniles of both species were subjected to acute and/or chronic sulfate exposures in soft water to compare sulfate sensitivity across age groups, and effective concentrations (EC)/lethal concentrations (LC) values were estimated. Mussels were individually exposed to allow relatively larger numbers of replicates per treatment. Chronic sulfate exposure significantly reduced growth, foot movement, and relative water content (RWC) in juvenile mussels of *M. margaritifera*. Mussels at younger stages were not necessarily more sensitive to sulfate. In the acute tests, LC50 of glochidia of *M. margaritifera* and *U. crassus* was 1301 and 857 mg/L, respectively. Chronic LC10 was 843 mg/L for 3-week-old *U. crassus* juveniles, 1051 mg/L for 7-week-old *M. margaritifera* juveniles, and 683 mg/L for 2-year-old *M. margaritifera* juveniles. True chronic Lowest Effective Concentration for 7-week-old *M. margaritifera* may be within the 95% interval of EC10 based on RWC (EC10 = 446 mg/L, 95%CI = 265–626 mg/L). Our study contributed to the understanding of sulfate toxicity to endangered freshwater mussel species in soft water.

## Introduction

The rise of major ion concentrations in freshwater ecosystems is a growing threat globally (Cañedo-Argüelles et al. 2013, 2019). Elevated sulfate concentrations in surface water result from, e.g., irrigation runoff, land clearing, mining, industrial wastewater, and road de-icing (Williams et al., (2000); Crosa et al. 2006; Muschal 2006; Bernhardt and Palmer 2011). High major ion concentrations in the ambient environment are often reported to result in biological degradation, inhibiting organismal growth and/or reproduction and possibly affecting population structure (Kennedy et al. 2005; Kunz et al. 2013; van Dam et al. 2014; Johnson et al. 2015, Hayward et al. 2022). Sulfate (SO_4_^2−^) is one of the most common major ions found in increasing levels in waterways with detrimental effects on freshwater ecosystems. Sulfate originates from sources such as runoffs from acid sulfate soils, sulfate pulping in paper mills, resource extraction, and fermented food processing (Lens et al., (1998); Nyberg et al. 2012; van Dam et al. 2014; Ekholm et al. 2020). For example, in the Central Appalachian streams in the U.S.A., sulfate-dominated wastewater caused by coal mining was found to increase sulfate concentration up to 40 times, resulting in severely impacted benthic macroinvertebrate diversity and abundance (Pond et al. 2008).

Mechanistic understanding of sulfate toxicity has been sparse, but the primary mode of action has been linked to osmotic stress caused by increases in salinity (Pond et al. 2008; Erickson et al. 2017; Scheibener et al. 2017). Major ion toxicity generally can be attributed to ion imbalance caused by enhanced levels of total dissolved solids (salinity/conductivity) in freshwater systems or by unusual compositions of ions (Goodf*e*llow et al. 2000). Conductivity was identified as an effective predictor of toxic effects from various industrial wastewaters, and meta-analyses have shown that salinity exerts a greater negative individual impact than other stressors such as temperature and toxicants (Pond et al. 2008; Kennedy et al. 2005; van Dam et al. 2014, Valesco et al. 2019). However, effluents with similar salinity levels but different specific major ion composition can cause varying toxic effects on freshwater organisms (Kunz et al. 2013, Scheibener et al. 2017). Even the same effluent can impose toxicity via different mechanisms, with some species being mainly affected by salinity and others by factors in addition to major ion concentrations (van Dam et al. 2014). Additionally, major ions differ in specific ion toxicity, with sulfate being ranked less toxic than potassium (K^+^) and bicarbonate (HCO_3_^−^) for freshwater animals such as *Daphnia* and minnows (*Pimephales promelas*) (Mount et al. 1997; Goodf*e*llow et al. 2000). Toxicity of major ions is also affected by co-occurring ions, with a notable example being calcium (Ca^2+^) ameliorating the toxicity of sodium (Na^+^) salts such as Na_2_SO_4_ (Soucek and Kennedy, 2005; Elphick et al. 2011; Mount et al. 2016). While the toxicity of potassium, magnesium (Mg^2+^), and calcium salts is most closely correlated with the cation’s chemical activity, in the case of sodium salts as Na_2_SO_4_, toxicity is mostly influenced by the anions and linked primarily to conductivity and osmotic stress (Mount *e*t al. 2016; Erikson et al. 2017). Previous acute toxicity tests on freshwater crustacean *Ceriodaphnia dubia* have demonstrated osmolarity as a useful exposure metric to reflect the toxicity of sodium salts (Erikson et al. 2017).

Like most aquatic invertebrates, Unionida species are hyperregulators in freshwater environments of lower salinity but behave as osmoconformers in higher salinities (Dietz et al. 2000; Griffith 2017). In low salinities, the organisms initiate energetically expensive mechanisms, such as downregulating membrane permeability to water and membrane carriers’ activity, to maintain a higher osmolality in their extracellular fluids (Rivera-Ingraham and Lignot 2017). When salinities in the surrounding medium increase, the organisms actively control ionic fluxes to maintain their extracellular fluids at the same as or lower osmolarities than the environment, thus becoming iso- or hypo-osmoregulators at high salinities (Rivera-Ingraham and Lignot 2017; Bradley 2009; Velasco et al. 2019). With their hemolymph osmolality ranked lowest among freshwater invertebrates (Burton 1983; McMahon and Bogan 2001), Unionid mussels may face heightened osmotic stress with enhanced energetic expenditure at sulfate-dominated increases in salinity. Mobilizing more energy for ion homeostasis leaves the animals with less resources for other functions such as growth and reproduction, and mortality ensues when vital functions are compromised over osmotic regulation demands (Kefford, 2018).

In the present study, we examined the toxicity of sulfate in the form of Na_2_SO_4_ on two endangered freshwater mussels, thick shelled river mussel (*Unio crassus*) and freshwater pearl mussel (*Margaritifera margaritifera*) (Unionida). In general sulfate transporters in aquatic species are poorly understood, and sulfate has been found to be relatively impermeant to gills of unionid mussels, freshwater fish, and crayfish (Garcia-Romeu and Maetz et al. 1964; Shaw 1960; de Renzis and Maetz 1973; MacMahon and Stuart 1989; Dietz et al. 2000; Griffith 2017). Sulfate uptake has been observed through whole body assays in some aquatic species, and the uptake may increase with environmental sulfate concentrations (Buchwalter et al. 2018; Griffith et al. 2020). Sub-lethal effects of sulfate exposure on freshwater mussels have been reported to reduce mussel growth, feeding, and post-feeding metabolism (Kennedy et al. 2003; Soucek 2007a; Hayward et al. 2022). Effective concentrations (ECx) and lethal concentrations (LCx) for various freshwater mussel species have also been previously produced through chronic and acute toxicity tests on mussel juveniles (Wang *e*t al. 2016, 2020). However, former studies have focused on sulfate toxicity in hard waters, and sulfate toxicity tests of mussels in soft waters have been lacking. Aquatic species in soft waters (CaCO_3_ < 40 mg/L) may exhibit enhanced vulnerability in face of anthropogenic salinization, and animal biotests in soft waters have suggested much lower EC/LC values than those conducted in hard or moderately hard waters (CaCO_3_ > 80 mg/L) (Soucek and Kennedy, 2005; Elphick et al. 2011; Mount *e*t al. 2016; Karjalainen et al. 2023). Given the heightened sensitivity to sulfate in organisms inhabiting softer waters, the remarkable ecological importance of freshwater mussels (Vaughn et al. 2008), and the global decline of freshwater mussel populations (Lopes*-*Lima et al. 2017; Ferreira-Rodríguez et al. 2019), it is imperative to fill such a gap in toxicity tests for mussels in soft water.

In ecotoxicological studies aiming to derive ECx, the demand to have a sufficient number of treatment levels to include the possible extreme concentrations often is met at the expense of replicate number per treatment level when animal use needs to be minimized (OECD 2006). In our study, a new methodology was proposed where mussels were exposed individually in test solutions in 6-well microplates. The use of microplates effectively separates the animals by placing one animal per well as one independent replicate in a treatment level, thus maximizing the number of replicates when test animals are limited, and when a high number of concentrations groups is desired for reliable ECx derivations. Placing multiple animals per replicate as suggested by ASTM standard guide (2022) may be risky when dead or unhealthy animals can negatively affect the rest of the animals in the same replicate while placing the animals individually safely removes such a risk factor (Karjalainen et al. 2021). Additionally, using one animal per replicate is particularly useful when behavioral activities need to be tracked separately and used as endpoint variables (Belamy et al. 2023). By exposing freshwater mussel glochidia and juveniles to sulfate in water-only solutions, we hypothesize that 1) in chronic tests, sulfate will negatively affect the survivability, growth, and movement of both freshwater mussels and relative water content of the mussels will be reduced, which will serve as a sensitive indicator of the effects of sulfate; 2) the mussels will show enhanced sulfate sensitivity (lower chronic EC10 or acute LC50) due to low water hardness compared with previous research on mussels in harder water; 3) younger stages of mussels are more sensitive to sulfate. This study aimed to contribute to the understanding of mussel sensitivity to sulfate in soft waters and generate EC and LC values for endangered mussels to aid in the development of environmental quality standards for freshwater ecosystems.

## Method

### Test organisms

Both mussel species *Unio crassus* and *Margaritifera margaritifera* prefer lotic conditions as their habitats and have a parasitic larval stage on suitable host fish species (Wächtl*e*r et al. 2001). The length of the parasitic period is temperature dependent and species-specific, varying from up to ten months in *M. margaritifera* to >1 month in *U. crassus* (Wächtl*e*r et al. 2001). During the parasitic stage, *M. margaritifera* glochidia, one of the smallest among Unionida freshwater mussels (glochidia length × width: 60 × 80 µm), can develop into juvenile mussels five times the initial size of the glochidia, whereas *U. crassus* juveniles do not grow in shell size (220 × 195 µm, Wächtl*e*r et al. 2001; Barnhart et al. 2008). Both species are listed as endangered in the IUCN Red List of Threatened Species, and their conservation status has been assessed as largely unfavorable at the EU biogeographical level (EEA 2013a; EEA 2013b; Lopes*-*Lima et al. 2014).

#### *Unio crassus* (thick shelled river mussel)

A total of 50 *Unio crassus* mussels (37 gravid females) were collected from Perniönjoki River in Southwest Finland in mid-May 2021 (permission to collect and maintain protected species, VARELY/2507/2021), at a river temperature of 14.6 °C. The mussels were transported in cooled, well-aerated water to Konnevesi Research Station, University of Jyväskylä, where they were maintained in flowing water over winter until mid-May 2022 when glochidia collection was carried out. The mussels received natural food from the incoming Lake Konnevesi water, and they were fed with commercial algal feeds Nanno 3600® and Shellfish Diet 1800® (Reed Mariculture Inc., USA). To stimulate glochidia release, water temperature was raised gradually from the winter temperature (1–2 °C) at the beginning of May to 14 °C by increasing the temperature by 1 °C every 24 h, and the mussels were transferred to plastic tanks for easier glochidia handling. Half of the water in plastic tanks was changed with aerated new Lake Konnevesi water every day, and the mussels were not fed during glochidia release. Mature glochidia were collected each day in the morning using a plastic tube which vacuumed water from the bottom of the plastic tanks into a bucket. In addition, 3-week-old juvenile *U. crassus* mussels from a rearing facility at the Mill of Kalborn (Luxembourg) were used in the short-term chronic 7 d toxicity test. The *U. crassus* mussels were originated from populations in the Our River bordering Luxembourg and Germany. Results by Wang et al. (2017) indicate that captivity-bred and wild juveniles of freshwater mussels demonstrate similar sensitivity in toxicity tests, thus justifying the use of captive-bred juveniles in the present study (Wang et al. 2017).

#### *Margaritifera margaritifera* (freshwater pearl mussel)

*M. margaritifera* glochidia and juveniles were collected from the artificial breeding program at the Konnevesi Research Station (Hyv*ä*rin*e*n et al. 2022). Adult mussels were collected from their original rivers, River Ähtävänjoki in Western Finland (permission to collect and maintain protected species, EPOELY/2278/2015) and River Luttojoki in Northern Finland (LAPELY/2252/2019). Brown trout (*Salmo trutta*) was used as the fish host for the glochidia (ESAVI/13097/2018). The adult and juvenile *M. margaritifera* were fed with the algal feeds Nanno 3600® and Shellfish 1800® (Reed Mariculture Inc., USA). Different age groups of mussels were fed according to the established feeding protocol at the local artificial breeding program during both cultivation and biotests. The feeding protocol was developed especially for *M. margaritifera* and was deemed to be sufficient for effective mussel growth (Hyv*ä*rin*e*n et al. 2022).

### Test water

The test water was prepared from filtered Lake Konnevesi (KV) water (tangential flow filtration Millipore Pellicon and Durapore GVPP 0.22 cassette, pore size 0.22 µm, Mg^2+^-Ca^+^ hardness 0.14 mmol/L i.e., 14 mg/L CaCO_3_ hardness). Sodium sulfate (Na_2_SO_4_, Merck, purity ≥ 99.0%) was dissolved into KV water to produce a gradient of nominal sulfate concentrations from 150 mg/L to 3500 mg/L. Each toxicity test had a different set of sulfate concentrations (Table 1). In addition to KV water, filtered River Kokemäenjoki water (KJ, tangential flow filtration Millipore Pellicon and Durapore GVPP 0.22 cassette, pore size 0.22 µm, Mg^2+^-Ca^+^ hardness 0.28 mmol/L i.e., hardness 28 mg/L CaCO3) was also used as a control treatment in two of the tests. KV and KJ water chemistry details were the same as in biotests from Karjalainen et al. (2023). The natural sulfate concentration of KV water varied from 4 to 6 mg/L and was 10 mg/L in KJ water (Table 1).

**Table 1:** General information of completed sodium sulfate (Na_2_SO_4_) toxicity tests with *U. crassus* and *M. margaritifera* mussels. Control waters were Konnevesi (KV, sulfate concentration in parentheses) and Kokemäenjoki (KJ) water. Temperature was presented as means ± standard deviations

Test type	24 h acute	7 d chronic	24 h acute	96 h acute	28 d chronic	28 d chronic
Species	*U. crassus*	*U. crassus*	*M. margaritifera*	*M. margaritifera*	*M. margaritifera*	*M. margaritifera*
Origin	River Perniönjoki	River Our	River Ähtävänjoki	River Luttojoki	River Ähtävänjoki	River Luttojoki
Start age	<24 h glochidia	3-week-old juveniles	<24 h glochidia	1-week-old juveniles	2-year-old juveniles	7-week-old juveniles
Exposure type	static	static-renewal	static	static	static-renewal	static-renewal
Light intensity	around 100 lux 16 h light	around 100 lux 16 h light	around 100 lux 16 h light	darkness 24 h	darkness 24 h	darkness 24 h
Temperature (°C)	16.4 ± 0.3	16.5 ± 0.2	16.2 ± 0.2	17.3 ± 0.2	17.3 ± 0.2	17.3 ± <0.1
pH	6.9–7.5	7.4–7.8	7.2–7.6	6.9–7.5	7.0–7.4	7.0–7.4
Feeding	no feeding	algal mix	no feeding	no feeding	algal mix	algal mix
Sulfate concentrations measured (mg/L)	KV (6), 280, 420, 560, 720, 790, 860, 1000, 1100, 1400	KV (5), 300, 593, 730, 1133, 1200, 1400, 1933	KV (5), 150, 300, 450, 593, 730, 825, 900, 1000, 1200, 1400, 1600	KV (4), KJ (10), 160, 300, 580, 1200, 1400, 1900, 3300	KV (4), 593, 890, 1200, 1333, 1667, 1967	KV (4), KJ (11), 307, 610, 750, 960, 1100, 1300, 1633
Replicate number per sulfate level	12	6	24	12–18	6	12

### Toxicity tests

#### Protocol modifications and justifications

The current ASTM standard guide (ASTM 2022) for freshwater mussel toxicity tests was developed mostly for mussels living in water that is moderately hard or hard (hardness >80 mg/L as CaCO_3_), with no mention of soft water mussel species *U. crassus* and *M. margaritifera* (hardness < 40 mg/L CaCO_3_). Thus, biotests on *U. crassus* and *M. margaritifera* were conducted with a new test methodology, and reasonable modifications to the standard are justified below.

The ASTM standard requires a minimum of 5 juvenile mussels per replicate in biotests, respectively, with at least 4 replicates per treatment and 200 mL of water per replicate (ASTM 2022). Due to the endangered status of both mussel species, no excessive amount of sample size was available for testing. New test methods for cases where only small sample sizes were available were thus developed. Lid-covered 6-well tissue culture microplates were used to expose juvenile mussels individually, namely, each replicate having only one animal to increase the number of replicates in each sulfate treatment level. The use of 6-well microplates with individually tested animals in bioassays was previously used by Karjalainen et al. (2021) who examined sulfate toxicity of whitefish embryos in the microplates. Individual placement of mussels in microplates was also utilized in the study of *M. margaritiera* locomotive behaviors to individually track mussel foot movement (Belamy *e*t al. 2023). In addition to increasing the possible number of replicates per treatment, the isolation of animals in the wells also prevents dead mussel from spreading water mold to living animals, which constitutes a considerable risk factor in chronic tests. Also, the glochidia tests were conducted in the microplates.

The ASTM standard also specifies age requirements for mussels used in toxicity tests, with 96 h acute tests using juvenile mussels younger than 10 days old, short-term chronic 7 d test using 1- to 3-week-old juvenile mussels, and chronic 28 d test using mussels up to 2 months old (ASTM 2022). All those age requirements were satisfied in the current study, with an extra age group of 2-year-old mussels used in a 28 d chronic test as a comparison. In *M. margaritifera*, a mussel species renowned for very slow growth rate and extreme longevity (over 200 years), 2-year-old mussels are still relatively young in its juvenile stage considering a sexually mature age of 10 to 15 years old (Skinner et al. 2003, Helama and Valovirta, 2008). In chronic tests, standard endpoints are survival and growth (ASTM 2022), which were documented in this study, when possible, while relative water content was also recorded for 7-week-old juveniles as an alternative endpoint considering that the slow growth rate may not reflect a concentration-response relationship. In addition, test temperatures for both mussels were lower than the ASTM standard requirement of 20–25 °C since the mussel species inhabit cooler water in their natural habitat (<20 °C). Compared to the standard illuminance of at least 100 lux (ASTM 2022), *M. margaritifera* juvenile tests were conducted in darkness following the protocol of the artificial breeding program at Konnevesi Research Station. Sulfate concentrations in each biotest were determined through range-finding preliminary tests.

#### Tests on *Unio crassus*

An acute 24 h sulfate exposure test was conducted with *U. crassus* glochidia less than 24 h old in May 2022. Before the experiment, glochidia viability was examined according to standard protocol with a drop of saturated NaCl solution, to which 100% of the glochidia responded by closing the valves (ASTM 2022). Glochidia were tested in lid-covered 6-well tissue culture microplates (VWR 6-well plate type 734–2717 6). Each well contained 10 mL test water and 10–12 glochidia individuals per replicate, with 12 replicates for each treatment level. Glochidia mortality (the number of glochidia that closed their shells upon the addition of a drop of saturated NaCl water / the total number of glochidia in each replicate) was recorded as the endpoint variable (Table 1).

A short chronic 7 d sulfate exposure test was conducted with 3-week-old *U. crassus* juveniles in June 2022. The experiment was conducted with the same microplate setting as mentioned above, but each well contained one individual juvenile in 10 mL of test water. A total of 48 animals were used in the experiment, with one animal per replicate and 6 replicates per treatment level. Before the experiment, all juveniles were confirmed to be alive by exhibiting active foot movement within 5 min of observation (ASTM 2022). The mussels were fed with algal mix, Nanno 3600® and Shellfish 1800® (Reed Mariculture Inc., USA) during the exposure. Mussel mortality was confirmed by open shells which did not close upon disturbance, and mortality was documented as the endpoint variable for LC calculations (Table 1). Wet and dry mass were not obtained for the 3-week-old *U. crassus* juveniles. Length measurements of all mussels were conducted at the start of the experiment and at the end for the surviving individuals.

#### Tests on Margaritifera margaritifera

An acute 24 h sulfate exposure test was conducted with *M. margaritifera* glochidia less than 24 h old in June 2022 (Table 1). Glochidia originated from a parental group of 65 adult mussels which were collected from River Ähtävänjoki. The adult mussels were kept in a maintenance tank, and the exact number of mussels which released the glochidia was not known. The glochidia test with 1 individual per replicate and 24 replicates per treatment level were carried out in lid-covered 24-well tissue culture microplates (VWR) and each well contained 2.5 mL of test water. 6 additional control replicates were prepared in a 6-well microplate with 8–10 glochidia per well in 10 mL test water. Glochidia viability was checked as described above to be 100% before the experiment.

Different age groups of *M. margaritifera* juveniles were used in toxicity tests (Table 1), and all juveniles were confirmed alive via active foot movement with 5 min of observation before experiments. In the 96 h acute exposure test conducted in July 2022, 149 individuals of 1-week-old juveniles, originated from populations in River Luttojoki, were subjected to different sulfate concentrations (one animal per replicate in each well of the microplate and 12 replicates per treatment level, with 18 replicates in the control group) in static exposure with no water change nor algal feeding. At the end of the experiment, foot movement of individual mussels in the microplates within 5 min of microscopic observation and mortality were used as endpoint variables for acute EC and LC calculations, separately. In the chronic 28 d tests for *M. margaritifera*, 7-week-old juveniles (one animal per replicate and 12 replicate per treatment level) and 2-year-old juveniles (one animal per replicate and 6 replicate per treatment level), originated from populations in River Luttojoki and Ähtävänjoki respectively, were subjected to toxicity tests in July and August 2021 (Table 1). The *M. margaritifera* juveniles were exposed in a static-renewal manner, where test water with algal additions (Nanno 3600 and Shellfish) was changed three times a week. Mortality was used as the endpoint for LC calculations. Dry mass, wet mass, and relative water content (RWC) were the end points for EC calculation. Mussels at the end of the chronic experiments were weighed for fresh mass and then oven-dried at 60 °C for 24 h for measurement of dry mass. For the 7-week-old juveniles, an extra group of 30 random individuals were weighed for wet and dry mass before the experiment. Relative water content (RWC), which was calculated as (Wet mass–Dry mass)/Wet mass, was also documented for each surviving mussel in the chronic tests. To obtain reliable wet mass measurements, each mussel was gently patted dry on the tissue paper for 2 s and then placed on the microscale for 20 s. The measurement process was standardized for each one of the surviving mussels to avoid unnecessary water loss from handling or measuring. Size variation was considered to be minimal as both age groups of juveniles were reared in the artificial breeding programs under the same conditions, and no length measurement was documented.

#### Test quality control

Depending on the availability of test organisms in each age group, each test had a different set of sulfate concentrations and a different number of replicates (Table 1). Sulfate concentrations were analyzed at the end of the tests for acute tests and at the start, middle, and end for chronic tests by an independent accredited laboratory KVVY (Tampere, Finland). Measured sulfate concentrations throughout all the toxicity tests were within ± 10% differences of nominal concentrations (Supporting information, Table S1). In all tests, oxygen levels in the microplate wells remained above 8 mg/L, which is higher than the minimum level of 4 mg/L mandated by the standard (ASTM 2022). Temperature and pH remained constant throughout the tests (Table 1). Water change was implemented three times a week in chronic tests with the aim of minimizing ammonia accumulation; ammonia was not measured but given the small mussel size, individual and separate placement of mussel in the wells, frequent water change, and the high control survival, ammonia levels should be minimal.

### Data analysis

For each toxicity test, 10%, 20%, and 50% effective concentrations (EC) or lethal concentrations (LC) with 95% confidence intervals were produced in R studio with the “drc” package (R version 4.3.1, Ritz et al. 2015). Based on Akaike information criterion (AIC) values and the obtainability of realistic EC/LC values with fitting curves, either two-parameter log-logistic function (LL.2) or three-parameter log-logistic function (LL.3, LL.3 u) was chosen for each analysis. In all the chronic tests, mussel fresh weight and dry weight failed to yield any concentration-response relation and relative water content was used as the endpoint variable to produce EC values for the 7-week-old *M. margaritifera* juveniles. Additionally, for comparison of mass-based variables for the 7-week-old *M. margaritifera* juveniles, Kruskal–Wallis H test and post-hoc Conover’s test (alpha = 0.05) with the step-down Benjamini-Hochberg test method were used (Dinno 2017). Due to the small sample size of 2-year-old *M. margaritifera* juveniles, only LC values were produced.

## Results

Both *U. crassus* tests met the toxicity test acceptability criteria, with the glochidia acute test having more than 90% control group survival and the 7 d chronic test having more than 80% control group survival (ASTM 2022). Based on mortality of *U. crassus*, acute LC50 of sulfate for *U. crassus* glochidia was 857 mg/L, and chronic LC10 for 3-week-old juvenile *U. crassus* was 843 mg/L (Table 2, concentration-response curves see Supplementary Information Figure S1). All alive 3-week-old *U. crassus* juveniles exhibited foot movement to some degree, with juveniles in lower concentrations being more active than the ones surviving from higher concentrations. *U. crassus* lengths did not differ between treatments before (Kruskal–Wallis *H* = 6.70, df = 5, *p* = 0.22) or after the treatment (Kruskal–Wallis *H* = 4.08, df = 3, *p* = 0.25)

**Table 2:** EC/LC values with 95% confidence interval (in parentheses) for sulfate (mg/L) for *Unio crassus* and *Margaritifera margaritifera* of different age groups in acute and chronic sodium sulfate exposures. RWC represents Relative water content

Test	Start age	endpoint	EC/LC10	EC/LC20	EC/LC50
*U. crassus*					
Acute (24 h)	<24 h glochidia	mortality	732 (690–774)	776 (742–810)	857 (833–881)
Chronic (7 d)	3-week-old	mortality	843 (NC–2153)	861 (NC–2163)	893 (NC–2204)
*M. margaritifera*					
Acute (24 h)	<24 h glochidia	mortality	878 (759–997)	1015 (912–1117)	1301 (1193–1410)
Acute (96 h)	7-day-old	foot movement	600 (383–818)	698 (500–897)	904 (749–1060)
Acute (96 h)	7-day-old	mortality	1035 (892–1179)	1122 (1003–1242)	1289 (1192–1386)
Chronic (28 d)	7-week-old	RWC	446 (265–626)	559 (396–722)	824 (689–960)
Chronic (28 d)	7-week-old	mortality	1051 (928–1174)	1135 (1031–1240)	1295 (1192–1399)
Chronic (28 d)	2-year-old	mortality	683 (471–896)	770 (578–961)	943 (781–1106)

*NC* Not calculable

In *M. margaritifera* tests, control group survival was 100% for 1-week-old, 7-week-old as well as 2-year-old juveniles. In the 96 h test on 1-week-old *M. margaritifera* juveniles, acute EC50 of sulfate was 904 mg/L with foot movement as the endpoint variable, while acute EC50 was 1289 mg/L with mortality as the endpoint variable (Table 2, concentration-response curves see Supplementary Information Fig. S1). In the 28 d test for 2-year-old *M. margaritifera*, only LC values were calculated. Chronic LC10 for 2-year-old *M. margaritifera* was 683 mg/L. In the glochidia test, the control group survival was 88%, slightly below the 90% test acceptability threshold. However, since survival in all 6 lower sulfate treatment groups of *M. margaritifera* glochidia from 150–825 mg/L was above 90%, the test was still included in the exposure-response analysis and LC50 was 1301 mg/L.

**Fig. 1:**
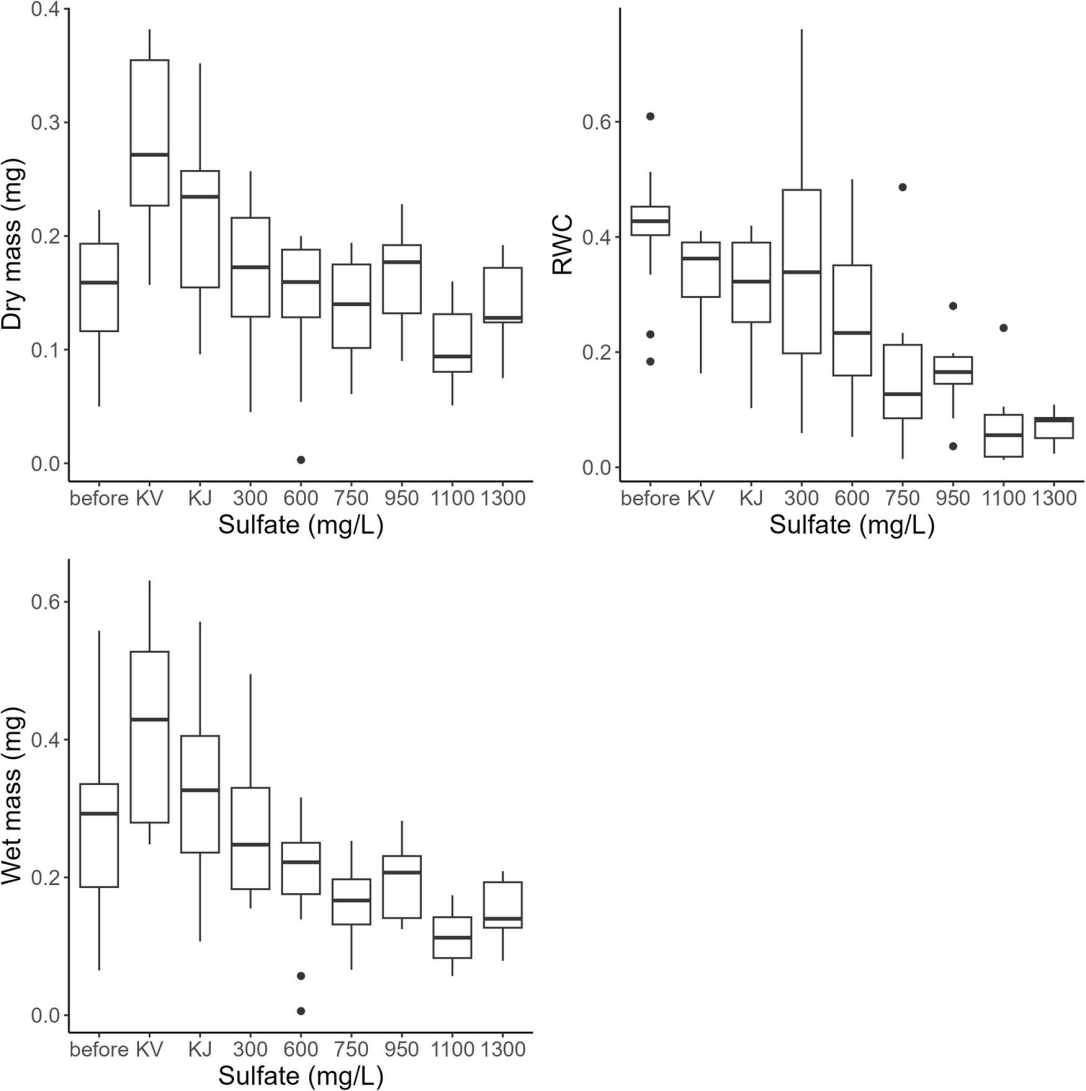
The effect of sulfate on 7-week-old *Margaritifera margaritifera* in terms of dry weight (left up), relative water content RWC (right up) and wet mass (left bottom). The “before” group indicates the starting level of the mussels before the experiment started. 12 replicates were exposed in each sulfate concentration, and individual measurements of surviving mussels were plotted

Chronic sulfate EC10 for 7-week-old *M. margaritifera* juveniles based on relative water content (RWC) was 446 mg/L, while chronic LC10 was 1051 mg/L, which was notably higher than the chronic LC10 of 2-year-old *M. margaritifera* juveniles (683 mg/L, Table 2, concentration-response curves see Supplementary Information Figure S1). In the 28 d test for 7-week-old juveniles of *M. margaritifera*, EC values could not be estimated based on dry mass or wet mass, since no concentration-response relationships were found based on those endpoint variables. However, sulfate significantly affected the dry mass of mussels (Kruskal–Wallis *H* = 33.95, df = 7, *p* < 0.05), the wet mass (Kruskal–Wallis *H* = 45.14, df = 7, *p* < 0.05), and relative water content (Kruskal–Wallis *H* = 39.89, df = 7, *p* < 0.05). KV control group showed significantly higher dry mass, wet mass, and RWC than all other sulfate-exposed groups from 300 mg/L to 1600 mg/L (Conover test, p < 0.05, Supplementary information Tables S2–S4). Also, *M. margaritifera* juveniles in the control were the only ones that exhibited significant growth over 28 days when compared to the dry mass at the beginning of the experiment, suggesting that the test setting and feeding arrangement were suitable for those juveniles (Fig. 1, Kruskal–Wallis *H* = 37.06, df = 8, *p* < 0.05). Wet mass was not significantly different between the control group and 300 mg/L treatment level, but they were significantly different from all the higher sulfate concentrations (Supplementary information Table S3). In terms of relative water content, KV control group (RWC 33.1%) was not significantly different from *M. margaritifera* juveniles in lower sulfate concentrations up to 600 mg/L (RWC 25.9%), but a significant difference could be seen when compared with those in sulfate levels higher than 750 mg/L (RWC 15.7%) (Fig. 1, Supplementary information Table S4).

In both 28 d tests on *M. margaritifera*, reduced valve and foot movement was observed. After the exposure, 7-week-old *M. margaritifera* juveniles showed active foot movement in sulfate concentrations up to 600 mg/L, while those that survived higher concentrations at the end of the experiment hardly moved their foot under 5 min observation. In 2-year-old *M. margaritifera* juveniles, foot movement was observed in the control group but not in the 600 mg/L treatment after the 28 d exposure.

## Discussion

Sulfate exposure had clear negative effects on the growth and survival of juvenile mussels. In 7-week-old *M. margaritifera* juveniles, sub-lethal sulfate exposure resulted in lower dry mass, wet mass, and relative water content. Since Unionid mussels accumulate sulfate rather slowly, the gill is considered to be relatively impermeable to the anion (Dietz *e*t al. 2000; Griffith 2017). Since sulfate cannot be effectively absorbed and eliminated at a high turnover rate like sodium, the elevation of sulfate-dominated salinity in the surrounding medium subjects the animals to enhanced osmotic stress, which can create metabolic burdens for the mussels as they actively expend energy mobilizing organic and/or inorganic osmolytes to regulate cell volume changes (Ruis and Souza 2008; Erickson et al. 2017; Scheib*e*ner et al. 2017). In our study, lower dry mass of sulfate-exposed 7-week-old *M. margaritifera* mussels compared to the control individuals suggested that balancing ambient salinity increases may have occurred at a high energetic cost, which possibly allocated energy from growth to ion homeostasis. A sulfate concentration as low as 300 mg/L was able to significantly affect 7-week-old *M. margaritifera* mussels’ growth, even though no concentration-related model could be fitted for growth variables at the end of the experiment. In addition, dry and wet mass of 2-year-old *M. margaritifera* juveniles and length of 3-week-old *U. crassus* juveniles were not detectably impacted by sulfate exposure and thus EC values could not be obtained. Soucek (2007a) also did not observe significant differences in Asian clam *Corbicula fluminea* shell dry weight or length after 28 days of sodium sulfate exposure at 1500 mg/L, though soft tissue dry weight showed significant reduction.

Limited growth in sulfate-affected juvenile mussels could be partly attributed to demonstrated continued valve closure and restricted foot movement across all tests. Mussels are known to close their valves in reaction to external stimuli and simultaneously reduce their filtration rate and oxygen consumption (Ortmann and Grieshaber 2003; Hartmann et al. 2015; Riisg*å*rd and Larsen 2015). In addition to valve movement depression, “avoidance behavior” has also been recorded in mussels under enhanced osmotic stress when the mussels close their shells tighter, leading to an elevated stress to adductor muscles and shortened open periods (Gainey 1978; Hartmann et al. 2015). In the case of sodium sulfate, 28 d chronic exposure has been reported to reduce filter feeding, post-feeding metabolism, and growth rates in *C. fluminea* (Soucek 2007a). In the present study, it is very likely that reduced shell and foot movement induced by sodium sulfate also limited mussel feeding and growth, especially for the 3-week-old *U. crassus* juveniles and 7-week-old *M. margaritifera* juveniles, whose underdeveloped gill apparatus called for active movement of foot for pedal feeding under normal circumstances (Schartum et al. 2017). Though physiological stress was not directly measured in the study, it is also possible that oxygen uptake and overall metabolism were reduced in osmotically stressed mussels as in other freshwater invertebrates exposed to higher salinity (Arn*é*r and Koivisto 1993; Soucek, 2007b). In short, sulfate exposure generated behavioral and physiological changes in mussels which negatively affected feeding and growth.

Based on RWC, a clear concentration-response relationship could be observed and EC10 with a relatively small confidence interval was obtained. RWC was not significantly different between 7-week-old *M. margaritifera* mussels in KV control group and those in lower sulfate concentrations, indicating relatively successful maintenance of tissue hydration up to 600 mg/L sulfate. However, the lower dry mass of sulfate affected *M. margaritifera* mussels suggested that water maintenance may have occurred at the cost of potential growth. In freshwater clam *C. fluminea*, muscle volume maintenance under hyperosmotic stress was also observed as the cells worked to minimize tissue volume changes induced by environmental salinity changes (Ruiz and Souza 2008). Since mussels in saline environments were able to maintain their water content at the cost of growth to some extent, this also shows that RWC may not be as sensitive as an endpoint as the growth endpoint variable dry weight. The weaker potency of RWC to accurately reflect toxic effects is plausible because, even though osmotic stress has been credited as the main mode of toxic action in sodium salts, it may not be the sole cause of toxicity (Mount et al. 2016; Erikson et al. 2017). Thus, the true lowest observed effect concentration (LOEC) of 7-week-old *M. margaritifera* mussels may be lower (below 300 mg/L sulfate based on dry weight comparisons) than the chronic EC10 based on RWC (446 mg/L), since the true LOEC should not only reflect tissue hydration changes but also compromised growth due to heightened osmotic regulation or other effects. It is possible that true LOEC is still within the 95% confidence interval of EC10 based on RWC (265–626 mg/L sulfate). However, in the case of long-living mussel species *M. margeritifera* where a chronic test has not been standardized, it is very possible that growth-based endpoint variables do not easily reflect concentration-response relationships during 28 d tests as described in other Unionida species. Thus, even though the use of RWC as an endpoint variable for producing EC values should be cautioned for likely being less sensitive than dry weight, in the case where mass-based growth endpoint variables could not yield concentration-response relationships from toxicity tests on slow-growing endangered species, RWC turns out useful in quantifying the sulfate sensitivity of *M. margaritifera* juvenile mussels in terms of the main toxic mechanism of sodium sulfate, namely osmotic stress. That no EC values could be acquired based on growth endpoints in our study could be due to 1) the limited number of surviving mussels in the tests, 2) that their growth changes were not distinguishable over the 7- to 28 d test periods due to the slow growth rate at low temperature (16–17 °C), which was typical of northern natural habitats. or 3) that extreme treatment levels below 300 mg/L were lacking.

Enhanced sensitivity to sulfate was observed in *M. margaritifera* juveniles inhabiting soft waters (CaCO3 < 40 mg/L), which showed relatively lower acute EC50 values and chronic LC10 values compared to other Unionida species tested in harder waters (CaCO3 > 80 mg/L). Wang *e*t al. (2017) reported that after 96 h sulfate exposure using hard water (CaCO3 = 100 mg/L), EC50 of 5 species of Unionida juveniles ranged from 1338 mg/L in three ridge mussels (*Amblema plicata*) to 2709 mg/L in paper pond shells (*Utterbackia imbecillis*), all higher than an acute EC50 of 689 mg/L in *M. margaritifera* in the present study. Similarly, in 28 d chronic sulfate experiments conducted by Wang *e*t al. (2016, 2020) using hard water (CaCO3 = 100 mg/L), LC10 values of 5- and 6-week-old juvenile mussels in hard water ranged between 1539 mg/L in rainbow mussels (*Villosa iris*) to 1645 mg/L in pink muckets (*Lampsilis abrupta*), clearly higher than a chronic LC10 of 7-week-old *M. margaritifera* juveniles (1051 mg/L) and the chronic LC10 of 2-year-old *M. margaritifera* juveniles (683 mg/L). Though various factors, such as differences in test water chemistry and mussel populations, could possibly explain the lower EC values of *M. margaritifera* in comparison with other Unionida species, lower water hardness in the KV water is likely to have played a major role in reinforcing sulfate toxicity, since calcium has been reported to ameliorate the effects of sulfate in multiple studies (Soucek and Kennedy, 2005; Elphick et al. 2011; Mount et al. 2016). The low acute EC50 and chronic LC10 values of *M. margaritifera* confirmed previous findings that freshwater organisms in softer waters are more sensitive to sulfate toxicity (Soucek and Kennedy 2005; Davies and Hall 2007; Elphick et al. 2011). This could possibly be due to the inhibition effects of calcium on gill permeability and passive diffusion, thus lowering energetic cost inflicted by osmoregulation (Soucek and Kennedy 2005). However, despite the lower acute EC and chronic LC values of *M. margaritifera*, the chronic EC10 value of *M. margaritifera* juveniles (446 mg/L, 95% CI 265–626 mg/L) did not exhibit enhanced sensitivity when compared with EC10 of other Unionida species from harder water, which ranged from 280 to 457 mg/L (Wang et al. 2016, 2020). This may be explained by our use of RWC as the endpoint variable rather than dry weight as in the previous studies since RWC is likely not as sensitive as dry weight.

Glochidia LC values showed that the larval stage was not more sensitive to sulfate toxicity than the juvenile stage. Glochidia LC50 values of both *U. crassus* (857 mg/L) and *M. margaritifera* (1301 mg/L) are comparable to acute and chronic EC/LC50 values for juveniles of both species, which ranged from 822 mg/L to 1295 mg/L. This observation also agrees with previous studies which have found that glochidia were similarly or even less sensitive to acute exposures of heavy metals and ammonia than juvenile mussels aged from 5 days to 2 months (Wang *e*t al. 2007, 2010). Noticeably, glochidia of *M. margaritifera* were more tolerable of sulfate toxicity than those of *U. crassus*. This could be partly explained by the size dimensions since *U. crassus* glochidia (length × width: 220 × 195 µm in size) has a much larger surface area than *M. margaritifera* glochidia (60 × 80 µm in size) (Wächtl*e*r et al. 2001). In exposure to a major ion like sulfate, glochidia surface area, rather than surface area to volume ratio, may be a more sensible factor to examine in this comparison, since mature glochidia tend to have their bivalves wide open, only periodically snapping close before attachment to host fish (Wächtl*e*r et al. 2001). In 24 h acute glochidia exposure to chloride in relatively hard water, species with smaller glochidia sizes (such as in Margaritiferidae) had higher EC50 values than many species with larger glochidia sizes, such as in Amblemini and Pleurobemini, though not all (Wang et al. 2017; Barnhart et al. 2008; Bringolf et al. 2022). The larger surface area of *U. crassus* glochidia potentially makes them more vulnerable to osmotic pressure caused by elevated sulfate levels, leading to enhanced sensitivity compared to the smaller glochidia of *M. margaritifera*. The test results should also be interpreted knowing that the *U. crassus* glochidia test and the *M. margaritifera* test had different experimental setups (12 replicates with 10 glochidia per replicate vs. 24 replicates with 1 glochidia per replicate, respectively). This difference in setup, together with the possible varying glochidia viability of the two species, may have resulted in the control survivorship of *M. margaritifera* glochidia being lower (88%) than *U. crassus* glochidia (>90%). For future biotests on *M. margaritifera*, 10 glochidia per replicate is recommended for toxicity tests in 6-well microplates when animal availability is not of concern. Individual placement of glochidia onto 24-well microplates can be time-consuming in practice due to the very small size of *M.margaritifera*.

While size may affect glochidia survival in sulfate exposures, age may also be a complicating factor in juvenile mussel sensitivity to sulfate toxicity, with younger juveniles being not necessarily more sensitive than older juveniles. Chronic LC10 in 7-week-old *M. margaritifera* mussels (1051 mg/L) was higher than that in 2-year-old juveniles of the same species (683 mg/L), though control groups in both tests were 100% alive with active foot movement observed at the end of the experiments. In addition, 7-week-old juveniles actively moved their foot in concentrations up to 600 mg/L while 2-year-olds hardly moved in the same concentration. It seems surprising that younger juveniles were more resistant to sulfate toxicity than older ones since Wang et al. (2010) reported higher acute EC50 values of Cadmium and Zinc for 6-month-old fat mucket (*Lampsilis siliquoidea*) juveniles than for 2-month-old juveniles. The difference in survivability between the 7-week-old and 2-year-old *M. margaritifera* juveniles could be explained by gill development. 2-year-old juveniles, which reached 4–5 mm in length, had transitioned to filter feeding thanks to their more mature gill apparatus, while the younger juveniles still relied on pedal feeding (Schartum et al. 2017). Even though mussels tend to close their valves in unfavorable conditions, the need for oxygen intake to avoid anaerobic metabolism necessitates incomplete closure or occasional opening (Riisg*å*rd and Larsen 2015). Even during the closed phase, tighter valve closure due to osmotic pressure may also fatigue abductor muscles in the long term, leading to even more incomplete shell closure (Hartmann et al. 2015). Thus, during such openings, more extensive gill surfaces in the 2-year-old juveniles likely subjected them to greater osmotic stress, leading to higher mortality and lower chronic LC values than the younger 7-week-old juveniles. Notably, the 7-week-old juveniles and the 2-year-old juveniles were originated from different populations in nature, which may also partly explain their differences in sulfate tolerance. Although LC10 of the 7-week-old *M. margaritifera* juveniles was much higher than the LC10 of the 2-year-olds, it needs to be noted that no EC values were calculated for the 2-year-olds due to the small sample size. EC values of the younger *M. margaritifera* juveniles could still be lower, but more research is warranted to answer this question. Our results suggested that younger mussels may not always be more sensitive than their older counterparts.

Due to the limited number of juveniles available for the tests, the test organisms of the two species were made up of different age groups, thus making impossible a direct comparison across species based on age. In addition, chronic test lengths varied, with *U. crassus* having a 7 d exposure and *M. margaritifera* having 28 d exposures. That *U. crassus* juveniles did not experience a longer experiment is due to concern that the juveniles would die of factors other than sulfate during their development throughout the 28 days, since high mortality of juveniles younger than 2 months was often observed in freshwater mussel culturing and testing (ASTM 2022). The 3-week-old *U. crassus* juveniles suit a short-term 7 d chronic test since the recommended age range for such tests is from 1 to 3 weeks old (Wang et al. 2021). Due to the small sample size of 3-week-old *U. crassus* juveniles, no reliable confidence intervals were obtained for LC calculations, thus leaving this test as a preliminary test that needs to be supported with follow-up research. However, given the endangered status of *U. crassus*, this biotest still provides useful information that can be cautiously interpreted in terms of sulfate effects across species. Survival data on the 7th day of the two 28 d tests can provide useful information on cross-species comparison, since both 7-week-old and 2-year-old *M. margaritifera* juveniles survived up to 1300 mg/L sulfate in 7 days, while 3-week-old *U. crassus* juveniles did not survive in 1100 mg/L sulfate. Based on 7 d survival, it seems 3-week-old *U. crassus* juveniles were more vulnerable to sulfate than *M. margaritifera* juveniles. 28 d sulfate exposure tests on older *U. crassus* juveniles are needed to enable more straightforward comparisons between juveniles of the two endangered species. Given their vulnerable early life stages, freshwater mussels are more sensitive than many commonly tested invertebrates, especially to toxicants such as copper and ammonia (Augspurger et al. 2003; Wang et al. 2007; March et al. 2007; Belamy et al. 2020). In sulfate exposures, mussel juveniles have been reported to exhibit higher sensitivity than midge larvae (*Chironomus dilutus*), amphipod juveniles (*Hyalella azteca*), rainbow trout eyed embryos (*Oncorhynchus mykiss*) but lower sensitivity than fathead minnow (*Pimephales promelas*) embryos (Wang et al. 2016, 2020). Even though the two endangered mussel species tested in the current study are not extremely sensitive to sulfate in the context of species sensitivity distribution (Karjalainen et al. 2023), their local rivers and catchment areas in southern Finland are potentially affected by wastewater from nearby forest operations, agricultural fields, manufacturing industries or mining activities with high sulfate loads, which can range from 1000 to 4000 mg/L (Ekholm et al. 2020; Oulasvirta et al. 2017; Runtti et al. 2018). It is important to note that aquatic animals may exhibit greater sulfate sensitivity in streams affected by coal mining wastewaters where sodium concentrations are often low, in contrast with standard test solutions spiked with sodium sulfate (Scheibener et al. 2017; Buchwalter et al. 2018). This is because sodium has also been identified as a modifier of acute sulfate toxicity, as higher sodium concentrations reduced sulfate uptake and improved mayfly larval survival in 96 h, suggesting an antagonistic relationship between sodium and sulfate transport (Scheibener et al. 2017).

In conclusion, our study shows that 1) sulfate exposure can lead to reduced growth, RWC and valve movement in juvenile freshwater mussels, 2) juvenile mussels inhabiting soft waters (CaCO3 < 40 mg/L) have lower acute EC and chronic LC values than those inhabiting harder waters, and 3) chronic sensitivity to sulfate can vary depending on the age of the juveniles, and younger mussels are not necessarily more sensitive. Testing the mussels individually in well plates as separate replicates maximized the use of the small sample size and prevented the spread of the infectious water mold from dead mussels, thus improving practical efficiency of the test methodology and statistical power of the test results. The use of different endpoints in concentration-response modeling also needs to be considered carefully and flexibly. Even though RWC did not turn out to be a more sensitive endpoint than dry weight, it still was a good alternative endpoint variable to produce EC10 with a 95% confidence interval when growth-based endpoint variables failed to produce any concentration-related response to sulfate exposure and provided insight into mussel physiological responses to sulfate. Most importantly, the present study contributes to the gap for ecotoxicology data of freshwater mussels in soft water, where organisms face enhanced threat to sulfate toxicity, and adds to the understanding of sulfate sensitivity of two important endangered mussel species *M. margaritifera* and *U. crassus*.

## Supplementary Information

Supplementary Information

## Data Availability

Data is provided within the manuscript or supplementary information files. Should any raw data files be needed in another format, they are available from the corresponding author upon reasonable request.
